# A Case Report: Management of Developmental Dysplasia With Cystic Lesions of the Left Hip Acetabular Bone With Pemberton Pelvis Osteotomy Planned With a 3D‐Printed Model

**DOI:** 10.1002/pdi3.70052

**Published:** 2026-05-26

**Authors:** Ting Li, Ke Sun, Jianglong Xu, Yushu Liu, Shengping Tang

**Affiliations:** ^1^ Department of Paediatric Surgery Qingxin District Hospital of Women and Children Healthcare Qingyuan Guangdong China; ^2^ Department of Orthopedics Shenzhen Children's Hospital Shenzhen Guangdong China

**Keywords:** 3D‐printed model, developmental dysplasia of the hip, dislocation of the hip joint, osteocystic lesion, Pemberton pelvis osteotomy

## 1 Background

Developmental dysplasia of the hip (DDH) is an abnormal development of the hip joint, causing the acetabulum not to match the femoral head and resulting mild subluxation to complete dislocation of the femoral head from the acetabulum. The lesions mainly involve the acetabulum, femoral head, joint capsule, and ligaments and muscles around the hip joint. They mainly manifest as abnormal matching and inclusion relationships between the acetabulum and femoral head. The mismatch between the acetabulum and the femoral head may accelerate degenerative changes in the joint, resulting in cartilage defects and/or subchondral cysts [1].

Pelvic osteotomy is a surgical treatment for DDH in ambulatory‐stage toddlers. The three primary osteotomy techniques are Salter, Dega, and Pemberton. Age may be an independent factor in the long‐term recovery of the hip joint after osteotomy. The pelvic bone and cartilage might be better shaped in younger patients (e.g., children), resulting in different hinge points during osteotomy, thus affecting the prognosis [2]. Suvorov et al. studied three surgical procedures using piglets and found that age is associated with the plasticity of bones and cartilage, which may affect the position and number of joints in the study. Experts advise against using Pemberton for children under 6 years of age [3]. Some researchers used 3D printing technology before surgery to simulate the affected hip joint and then performed osteotomy to reduce the impact on bone and cartilage tissue. This technique can simulate the special anatomical structure of children and provide specific intervention measures to reduce the operation time and long‐term impact [4, 5]. In surgical procedures, the advantages offered by 3D printing technology include reduced operating room time, enhanced visualization, decreased radiation exposure, improved cost‐effectiveness, and better clinical outcomes through customized surgical instruments [6, 7, 8]. 3D‐printing technology has revolutionized the field of orthopedics, playing a pivotal role particularly in pre‐operative planning and providing solutions for the repair and reconstruction of extensive bone defects [9]. Children with DDH undergoing proximal femoral osteotomy with 3D‐printed osteotomy guide can benefit from a simplified surgical process, shorter operative time, reduced intraoperative blood loss, and decreased radiation exposure. This technology demonstrates significant clinical value [10].

Pemberton osteotomy presents two main problems in congenital dislocation or subluxation of the hip joint in older children. One is that the acetabulum is shallow and inclined anterolateral; the other is that the acetabulum does not match the femoral head. Therefore, the indications for Pemberton pelvis osteotomy include hip dislocation or subluxation, especially for the shallow flat acetabulum in children aged 1 to 14 years [11, 12].

## 2 Case Presentation

### 2.1 History of Presenting Illness

**FIGURE 1:**
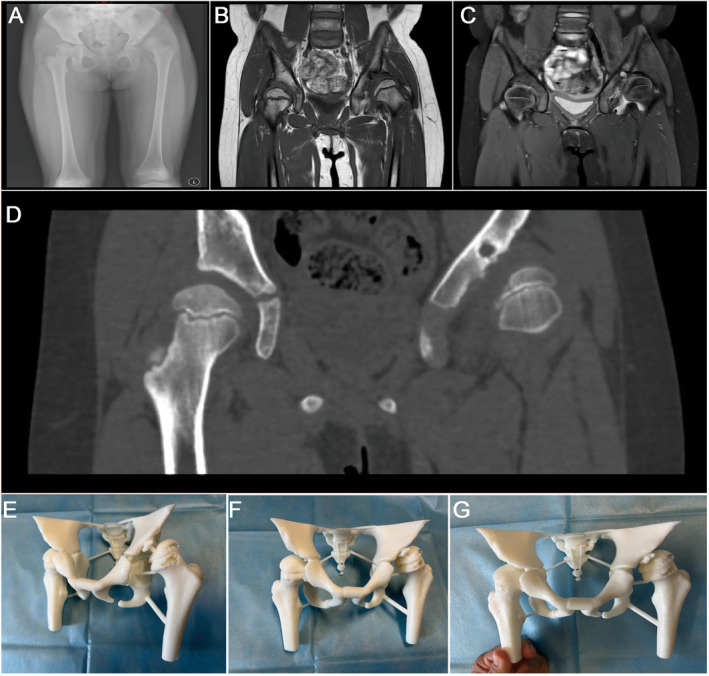
Preoperative imaging and 3D‐printed models. (A) Preoperative anteroposterior radiographs of the pelvis; magnetic resonance imaging of the osteocystic lesion (B, C); computed tomography of the hip joint osteocytic lesion (D); and 3D‐printed one‐to‐one models (E–G)

A 9‐year‐old girl presented with clinically abnormal gait for 1 year and left hip pain. On examination, internal rotation and abduction of the hip were restricted, with mild shortening on the left side. The left lower limb was about 1.5 cm shorter than the right lower limb and Allis (+). The pelvic radiograph showed subluxation and partial deformation of the left femoral head with a shallow dysplastic acetabulum and a circular bone defect of the left iliac crest. Further magnetic resonance imaging (MRI) examination revealed a cystic lesion about 21 mm × 15 mm × 24 mm (left/right diameter × front/back diameter × upper/lower diameter) (Figure 1).

A review of the patient's medical history showed that the patient had been treated for frequent toe‐walking and falls when she began to walk stradily at around 1‐year‐age. At that time, the doctor only did a physical examination and ignored it. The child began to learn dance at age four until she went to the pediatric orthopedic clinic complaining of leg pain. After a physical examination (rest and massage) and radiological examination, she was diagnosed with DDH.

Prior to the planned surgery, computed tomography (CT) revealed subplanar osteocystic lesions of the affected acetabular joint, which increased the difficulty of surgery. After team discussion, we decided to use preoperative 3D‐printing technology to construct the affected side model and simulate the implementation of Pemberton osteotomy. In DDH, the use of Pemberton osteotomy combined with proximal femoral osteotomy can maintain concentric reduction of the femoral head and acetabulum, emphasize joint capsule tightening sutures, and reduce the incidence of femoral head necrosis [13]. For older children with DDH, the combination of Pemberton osteotomy and femoral shortening osteotomy demonstrates superior advantages in reducing postoperative complications and promoting functional recovery, along with achieving higher postoperative satisfaction rates [14].

The child's parents agreed to submit data concerning the case for publication, and the child herself also consented.

### 2.2 Surgical Treatment

We selected Pemberton osteotomy after performing simulated osteotomy on the 3D one‐to‐one model of the child's pelvis structure (Figure 1E–G). We selected a Smith‒Petersen approach incision with a length of approximately 10 cm for the left hip joint. The lateral joint capsule was detached from the gluteus minor muscle to expose the joint capsule. After external rotation of the hip joint, we exposed the iliopsoas muscle, and the critical part was severed. In the fully exposed anterior and medial part of the hip capsule, the joint capsule was incised with a T‐shaped incision, and part of the long lateral joint capsule was excised. We took triangular bone pieces from the iliac crest. Arcuate osteotomy began along the inner and outer plates of the iliac crest approximately 1 cm above the anterior inferior iliac spine. The osteotomy line of the internal iliac plate ran from the anterior inferior iliac spine to the midpoint of the iliopectineal cartilaginous line. The osteotomy line of the external iliac plate ran from the anterior inferior iliac spine to the center of the iliac cartilage line. The broken end of the osteotomy was extended and flipped using the Y‐shaped cartilage as a hinge. The iliac triangular bone block was inserted and fixed with a 2.0 Kirschner wire. Bedside radiographs showed a satisfactory reduction of the pelvic osteotomy and hip joint. Intraoperative bleeding was approximately 200 mL.

### 2.3 Postoperative Course

**FIGURE 2:**
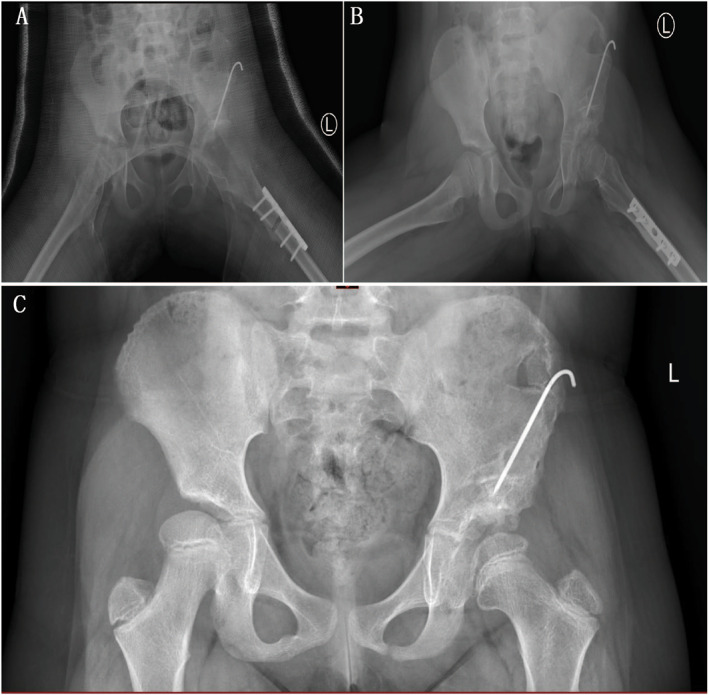
Anteroposterior radiographs of the pelvis during follow‐up after Pemberton osteotomy. Three days after surgery (A) 3 months after surgery (B), and 6 months after surgery (C).

Due to children's particularity and clinical experience, deep vein thrombosis rarely occurs in children. The incidence of Venous Thromboembolism in children at the population level is very low, reported to be 0.07 to 0.14 per 10,000 children [15, 16, 17]. Nursing staff instructed parents of the patient on post‐operative precautions and rehabilitation methods. Three days after surgery of the patient, an X‐ray examination was performed, and the results are shown in Figure 2A.

At 8 weeks, the patient gradually resumed movement after the hip herbalism was removed. Twelve weeks postoperatively, the patient demonstrated significant satisfaction with the surgical outcomes and reported no significant pain in either the hip joint or inguinal region. The child achieved independent ambulation with the aid of bilateral crutches. Physical examination revealed a significantly improved range of motion in the left hip joint compared to the preoperative assessment. X‐ray (Figure 2B) examination of both hip joints in frog position shows bilateral femoral head ossification center opposite bilateral acetabulum. The left and right acetabular angles were about 27° and 19°, respectively. The left acetabulum was still shallow.

At the 6‐month visit, children usually pay attention to exercise and activities. She gradually lost her crutches. However, the child still had a left lower limb shorter than the right side by approximately 1.5 cm. Her X‐rays demonstrated evidence that the acetabulum and femoral head matched better than before and that there was good femoral head coverage (Figure 2C).

**FIGURE 3:**
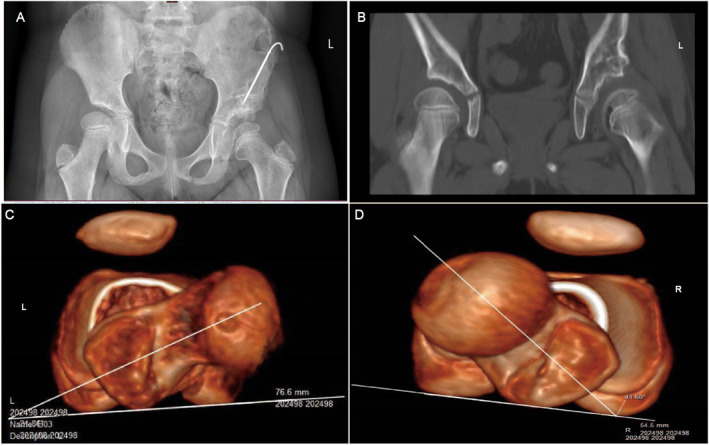
Follow‐up images, 1 year after surgery: anteroposterior radiographs of the pelvis (A) and computed tomography scan of the hip joint showed no lesions (B); 3D reconstruction of the left/right acetabular angle (C, D).

At 1 year after surgery, the child's parents remained delighted. She was pain‐free, with normal range of motion in the left hip joint and normal gait pattern, maintaining a high level of functional capacity. The Kirschner wires and plates were removed after the patient was re‐hospitalized. The left and right acetabular angles were about 10° and 15°, respectively. The left acetabulum was opposite the center of the femoral head (Figure 3A).

**FIGURE 4:**
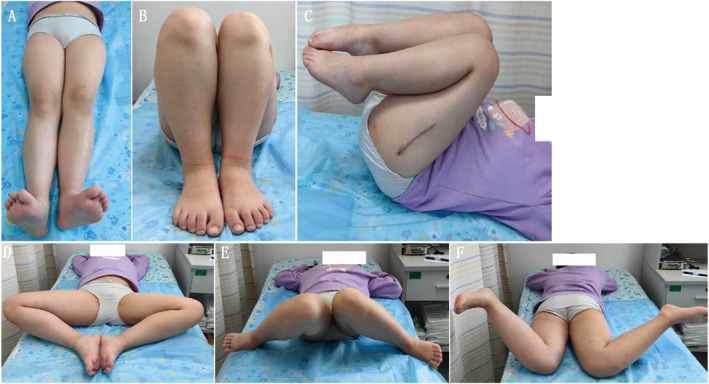
Physical examination, 1 year after surgery. Supine position (A), Allis(+) (B), flexion position(C), external booth (D), internal spin position (E), and pronation in the prone position (F)

During follow‐up, we found that the osteocapsular lesion below the surface of the left acetabular joint was gradually reduced. One week after the removal of the internal plate, a CT examination revealed that the osteocytes disappeared below the articular surface of the left hip joint (Figure 3B). Physical examination showed that the patient's internal rotation, external rotation, abduction, and flexion functions were better than before surgery, but Allis (+) was present. The child's left and right lower limbs were shortened by approximately 1.5 cm, which can be compensated by adding extra support to the insole (Figure 4).

## 3 Discussion and Conclusions

In patients with neglected DDH, bone cystic lesions below the acetabular closure surface have not yet been clearly reported. In the treatment of DDH, the most suitable surgical modality is Pemberton pelvis osteotomy for hip dislocation or subluxation. In this case, the choice of surgical procedure presented a challenge to surgeons.

To solve this problem and reduce intraoperative accidents, we adopted the concept of using 3D models before surgery. Preoperation on the model can predict the intraoperative situation in advance and provide an operation in possibly the safest and most effective way. In this particular case, external fixation of the Pemberton pelvis osteotomy and hip was performed. We performed a CT examination after the child returned to normal movement and found that the osteocystic lesion had decreased in size under the hip joint surface. This suggests that osteocystic lesions may occur due to changes in the direction of hip stress. However, after Pemberton pelvis osteotomy surgery, some children have bone or acetabular cysts [18]. In the study by Vasyl Suvorov et al., it was reported that this may be related to the age of the patient [3]. Children have a better ability to shape their bones and cartilage than adults. Can it be understood that the acetabulum and femoral head of children with DDH can better match each other after surgery due to the strong shaping ability of bone and cartilage? Findings from animal models suggest that the hinge points of osteotomy in older people are different from those in younger people. Could this finding also explain one of the reasons for the reduction of osteocysts in this child after surgery [3, 19, 20, 21, 22]? These studies and cases give pediatric surgeons much inspiration when dealing with complex or older children with DDH.

In the case presented in our study, bone was taken from the iliac crest and implanted into the osteotomy segment to reduce rejection due to the allogeneic bone [23]. Chen et al. reported a special case of DDH in which they found semi‐free intraarticular osteoclast cartilage tissue in the affected hip. Interestingly, immunohistochemical analysis revealed the expression of type I collagen, indicating that the formation of chondroid tissue originates from tear injuries of the articular cartilage. Such lesions should be classified as one of the pathological types of DDH [24]. It has a typical ball‐and‐socket shape from embryonic development and becomes shallow at birth. The surgeon uses the acetabular index (AI) to assess the extent of that the acetabulum covers the femoral head. In order to obtain better movement ability of children after surgery, it is necessary to consider the problem of anterior inclination during surgery to obtain a satisfactory AI [25]. As surgeons, we need to be very careful about how we operate on different children. We need to remember that the safety and recovery of the child is the most important thing. In Lucchesi et al.'s long‐term follow‐up of patients with DDH wih a mean follow‐up of 23.5 years, no total hip replacement (THR) was observed. Notably, a substantial proportion of patients required THR much later: 10.2% between 30 and 40 years and 35.6% more than 40 years after the surgery [26]. For patients with late‐detected DDH, the poor prognosis appears to be attributed to the pathology itself rather than the treatment modality. Age was an independent factor affecting the prognosis and complications of DDH [27]. The earlier DDH is detected, the more effective the treatment. This requires a country to start screening earlier and reduce the DDH of late treatment due to neglect.

In summary, this report presents a case of a neglected DDH with a subchondral cyst lesion in the acetabulum, which was successfully treated with Pemberton acetabuloplasty. This surgical approach serves as an effective joint‐preservation treatment option that can be combined with other procedures, demonstrating promising short‐term outcomes in the management of such cases.

## Author Contributions

Ting Li collected data and wrote papers. Ke Sun and Yushu Liu carried out the surgical plan. Shengping Tang provided funding. Jianglong Xu made pictures.

## Funding

This work was partially funded by Exporation on the Application of Case‐Based Learning, Multi‐Disciplinary Team, and High‐Quality Course System Construction in Standardized Residency Training of Pediatric Surgery (No. 25).

## Ethics Statement

This case report has been approved by the child's guardian, and has been reviewed and approved by the unit's ethics committee of Shenzhen Children’s Hospital (No. 2023002).

## Consent

Written informed consent was obtained from the patient's legal guardian for the publication of this case report with any accompanying images. The patient was informed that data concerning the case would be submitted for publication, and she provided consent.

## Conflicts of Interest

The authors declare no conflicts of interest.

## Data Availability

The data used or analyzed during the current study are available from the corresponding author upon reasonable request.
